# Population-based epidemiological data of follicular lymphoma in Poland: 15 years of observation

**DOI:** 10.1038/s41598-020-71579-6

**Published:** 2020-09-03

**Authors:** Anna Szumera-Ciećkiewicz, Urszula Wojciechowska, Joanna Didkowska, Jan Poleszczuk, Grzegorz Rymkiewicz, Ewa Paszkiewicz-Kozik, Kamil Sokół, Monika Prochorec-Sobieszek, Jan Walewski

**Affiliations:** 1 Department of Pathology and Laboratory Diagnostics, Maria Sklodowska-Curie National Research Institute of Oncology, W.K. Roentgen 5, 02-781 Warsaw, Poland; 2 Department of Diagnostic Hematology, Institute of Hematology and Transfusion Medicine, Warsaw, Poland; 3 Polish National Cancer Registry, Maria Sklodowska-Curie National Research Institute of Oncology, Warsaw, Poland; 4 Nalecz Institute of Biocybernetics and Biomedical Engineering, Polish Academy of Sciences, Warsaw, Poland; 5 Department of Computational Oncology, Maria Sklodowska-Curie National Research Institute of Oncology, Warsaw, Poland; 6 Department of Lymphoid Malignancies, Maria Sklodowska-Curie National Research Institute of Oncology, Warsaw, Poland

**Keywords:** B-cell lymphoma, Cancer epidemiology

## Abstract

Available epidemiological reports on follicular lymphoma (FL) often highlight a significant discrepancy between its high and low incidence rates in Western and Eastern Europe, respectively. The reasons behind that difference are not fully understood, but underreporting is typically presumed as one of the main factors. This study aimed to assess FL epidemiology in Poland based on 2000–2014 data from the Polish National Cancer Registry, which has 100% population coverage and over 90% completeness of the registration. All cases were coded according to ICD-10 and ICD-O-3 recommendations. The total number of registered FL cases was 3,928 with crude (CR) and standardized (SR) incidence rates of 0.72/10^5^ and 0.87/10^5^, respectively. The median age of FL diagnosis was 61 years, with the male to female incidence ratio of 1.06. The distribution of morphological types of FL: not otherwise specified (NOS), grades 1, 2, or 3 were 72.58, 4.81, 12.88, and 9.73%, respectively. Among all reported mature B-cell non-Hodgkin lymphomas, FL was ranked the fourth in incidence, just after chronic lymphocytic leukemia/small lymphocytic lymphoma (CR 3.62/10^5^, SR 4.99/10^5^), plasma cell neoplasms (CR 3.78/10^5^, SR 4.97/10^5^) and diffuse B-cell lymphoma, NOS (CR 2.13/10^5^, SR 2.65/10^5^). The systematic increase in FL incidence among females was observed. Our study confirms a lower FL incidence rate in Poland as compared to other European countries. Moreover, as our analysis was based on a registry with high data completeness, it provides evidence that reasons other than underreporting are responsible for FL incidence discrepancies between Eastern and Western Europe.

## Introduction

Follicular lymphoma (FL) is one of the most common non-Hodgkin’s lymphomas (NHLs) and its incidence varies between geographical regions. In Western Europe and US FL accounts for 20–40% of all NHLs, whereas in Eastern Europe, Asia, and developing countries, its prevalence is about threefold lower. FL affects mostly white adults of a median age of sixty, with a slightly higher incidence rate in females^1^. There is an upward trend in the incidence of NHLs in Western countries, which can be attributed mainly to the increased incidence of FL^2^. According to data from the Polish histopathological registry of lymphomas, FL accounted for less than 5% of all diagnoses reported in the 2007–2012 period^3^.

One of the main risk factors predisposing to FL is exposition to high doses of pesticides and herbicides, which may induce *BCL2* gene translocation t(14;18)(q32;q21)^4^. FL heterogeneity poses a diagnostic and therapeutical challenge, from early indolent lymphoma to aggressive transformation into therapy-resistant diffuse large B-cell lymphoma, not otherwise specified (DLBCL, NOS). FL is composed of germinal follicle center B-cells containing centrocytes and centroblasts that almost always present focal follicular growth pattern^4,5^. An absolute number of centroblasts per high-power microscopic field should be evaluated by a pathologist to classify FL into the histological grading system according to the International Classification of Diseases-Oncology (ICD-O-3) codes. The majority of cases are categorized as low-grade (grade 1/2) lymphomas, while high-grade (grade 3) subtypes are being reported in about 10–20% of patients. The frequency of grade 3A versus 3B has not been deeply studied^6^. The pure grade 3B is rare and contains diffuse areas composed of centroblasts^4,7^. Biologically, it is more closely related to DLBCL, NOS, and manifests clinically with higher short-term mortality and intermittent remissions after chemotherapy^8^. The cases without grade specification (not-otherwise specified, NOS) are classified together with pediatric-type FL. In addition, the revised 4^th^ edition of the World Health Organization (WHO) classification of Tumours of Haematopoietic and Lymphoid Tissues separates in situ follicular neoplasia (ISFN), formerly referred to as follicular lymphoma in situ. In reactive lymph nodes, ISFN is found in about 3% of cases and is associated with a low rate of clinical progression^9^.

There are only a few published epidemiological studies on FL in Poland^3^. However, the current consensus is almost exclusively based on the data published in the HAEMACARE project for Europe, which covered only about 10% of the Poland population (data from 3 local registries)^1^. Here, we analyze the FL incidence and mortality in Poland based on the data from the Polish National Cancer Registry (NCR), which has 100% population coverage and over 90% completeness of the registration. We report the first long-term observational data being under histopathological supervision. The FL trends against other B-cell NHLs and age group-specific rates are also investigated.

## Results

**Figure 1:**
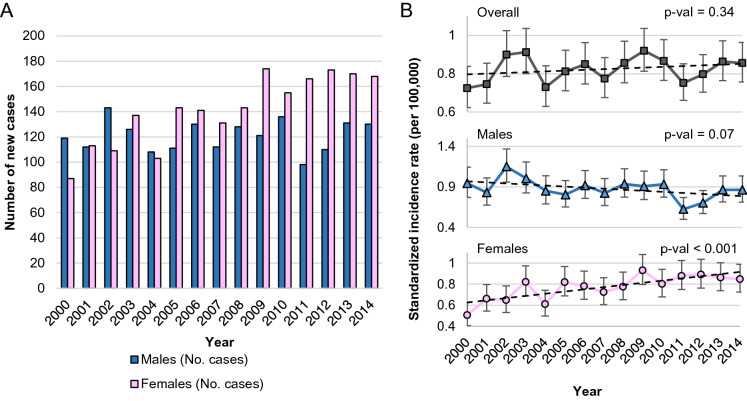
Follicular lymphoma number of new cases and standardized incidence rate, Poland 2000–2014.

Among all of the new cancer cases reported to the NCR in the years 2000–2014, FL accounted for 6.3% of all mature B-cell NHLs, with 5.6% in the male and 6.9% in the female populations, respectively. FL was the fourth most common mature B-cell NHLs in Poland (see Table 1, Supplementary Fig. S1 online). The number of reported FL cases increased from 209 in 2000 to 298 cases in 2014, with the upward tendency more strongly marked in the female population (see Fig. 1A). We observed no significant trend in the standardized overall FL incidence rates (P-val = 0.34), a borderline significant decrease in the standardized FL incidence rate among males (P-val = 0.07), and a significant increase in the standardized incidence among females (P-val < 0.001) (see Fig. 1B). The incidence rate for the studied period increased by about 50% in the female population, from approximately 0.6/10^5^ at the beginning of the twenty-first century to 0.9/10^5^ in 2014.

**Table 1:** Mature B-cell NHLs incidence including ICD-O-3 subtyping, Poland 2000–2014.

Mature B-cell NHLs group	ICD-O-3 code	ICD-O-3 description	MALES					FEMALES					ALL				
			Cases	CR	95% CI	SR	95% CI	Cases	CR	95% CI	SR	95% CI	Cases	CR	95% CI	SR	95% CI
CLL/SLL			11,887	4.28	4.21–4.36	6.55	6.43–6.68	8,906	3.01	2.94–3.07	3.43	3.36–3.50	20,793	3.62	3.57–3.67	4.99	4.92–5.06
	9,670	Malignant lymphoma, small B-cell lymphocytic, NOS	1,375	0.50	0.47–0.52	0.72	0.68–0.76	987	0.35	0.33–0.38	0.39	0.37–0.42	2,362	0.42	0.41–0.44	0.56	0.53–0.58
	9,823	B-cell chronic lymphocytic leukemia/small lymphocytic lymphoma	10,512	3.79	3.71–3.86	5.84	5.72–5.95	7,919	2.67	2.61–2.73	3.06	2.99–3.13	18,431	3.21	3.17–3.26	4.45	4.38–4.51
Immunoproliferative diseases			686	0.26	0.24–0.28	0.40	0.37–0.43	615	0.23	0.21–0.25	0.26	0.24–0.28	1,301	0.25	0.23–0.26	0.33	0.31–0.35
	9,671	Malignant lymphoma, lymphoplasmacytic	190	0.08	0.07–0.09	0.11	0.09–0.13	144	0.06	0.05–0.07	0.06	0.05–0.08	334	0.07	0.06–0.08	0.09	0.08–0.10
	9,760	Immunoproliferative disease, NOS	130	0.06	0.05–0.08	0.10	0.08–0.11	137	0.06	0.05–0.07	0.07	0.06–0.08	267	0.06	0.05–0.07	0.08	0.07–0.09
	9,761	Waldenström macroglobulinemia	363	0.22	0.20–0.25	0.32	0.28–0.35	326	0.14	0.13–0.16	0.16	0.14–0.18	689	0.18	0.16–0.19	0.23	0.21–0.25
	9,762	Heavy chain disease, NOS	3	0.01	0.00–0.02	0.01	0.00–0.02	8	0.01	0.00–0.02	0.01	0.00–0.02	11	0.01	0.00–0.01	0.01	0.00–0.01
Mantle cell lymphoma	9,673	Mantle cell lymphoma	1,397	0.63	0.60–0.66	0.91	0.86–0.96	679	0.29	0.27–0.32	0.33	0.31–0.36	2076	0.46	0.44–0.48	0.63	0.60–0.65
Follicular lymphoma			1815	0.65	0.62–0.68	0.87	0.83–0.91	2,113	0.79	0.76–0.83	0.87	0.83–0.90	3,928	0.72	0.70–0.75	0.87	0.84–0.90
	9,690	Follicular lymphoma, NOS	1,363	0.49	0.47–0.52	0.66	0.62–0.70	1,488	0.56	0.53–0.59	0.61	0.58–0.64	2,851	0.52	0.51–0.54	0.64	0.61–0.66
	9,691	Follicular lymphoma, grade 2	78	0.04	0.03–0.05	0.04	0.03–0.06	111	0.05	0.04–0.06	0.05	0.04–0.06	189	0.04	0.04–0.05	0.05	0.04–0.06
	9,695	Follicular lymphoma, grade 1	214	0.09	0.08–0.10	0.11	0.10–0.13	292	0.13	0.11–0.14	0.14	0.12–0.15	506	0.11	0.10–0.12	0.12	0.11–0.14
	9,698	Follicular lymphoma, grade 3	160	0.06	0.06–0.08	0.08	0.07–0.10	222	0.09	0.08–0.10	0.10	0.09–0.11	382	0.08	0.07–0.08	0.09	0.08–0.10
Diffuse B-cell Lymphoma			5,857	2.11	2.06–2.16	2.91	2.83–2.99	6,395	2.16	2.11–2.21	2.39	2.33–2.45	12,252	2.13	2.10–2.17	2.65	2.60–2.70
	9,675	Malignant lymphoma, mixed small and large cell, diffuse (obsolete)	107	0.05	0.04–0.06	0.06	0.05–0.07	100	0.05	0.04–0.07	0.06	0.05–0.07	207	0.05	0.04–0.06	0.06	0.05–0.07
	9,679	Mediastinal large B-cell lymphoma	41	0.02	0.01–0.02	0.02	0.01–0.02	48	0.02	0.01–0.03	0.02	0.01–0.02	89	0.02	0.01–0.02	0.02	0.01–0.02
	9,680	Malignant lymphoma, large B-cell, diffuse, NOS	5,613	2.02	1.97–2.08	2.79	2.72–2.87	6,159	2.08	2.03–2.13	2.30	2.24–2.36	11,772	2.05	2.01–2.09	2.55	2.50–2.60
	9,684	Malignant lymphoma, large B-cell, diffuse, immunoblastic, NOS	96	0.04	0.03–0.05	0.05	0.04–0.07	88	0.04	0.03–0.05	0.04	0.03–0.05	184	0.04	0.03–0.04	0.05	0.04–0.06
Burkitt lymphoma/leukemia			522	0.19	0.17–0.20	0.19	0.17–0.21	227	0.08	0.07–0.09	0.08	0.07–0.09	749	0.13	0.12–0.14	0.13	0.12–0.14
	9,687	Burkitt lymphoma, NOS	484	0.17	0.16–0.19	0.18	0.16–0.19	196	0.07	0.06–0.08	0.07	0.06–0.08	680	0.12	0.11–0.13	0.12	0.11–0.13
	9,826	Burkitt cell leukemia	38	0.02	0.01–0.02	0.02	0.01–0.02	31	0.01	0.01–0.02	0.01	0.01–0.02	69	0.01	0.01–0.02	0.01	0.01–0.02
Marginal zone lymphoma			662	0.27	0.25–0.29	0.38	0.35–0.41	814	0.33	0.31–0.35	0.36	0.34–0.39	1,476	0.30	0.29–0.32	0.37	0.35–0.39
	9,689	Splenic marginal zone B-cell lymphoma	37	0.03	0.02–0.04	0.04	0.03–0.05	36	0.03	0.02–0.04	0.03	0.02–0.04	73	0.03	0.02–0.04	0.03	0.03–0.04
	9,699	Marginal zone B-cell lymphoma, NOS/mucosa-associated lymphoid tissue lymphoma	623	0.26	0.24–0.28	0.36	0.33–0.39	776	0.32	0.29–0.34	0.35	0.32–0.37	1,399	0.29	0.27–0.30	0.35	0.33–0.37
	9,764	Immunoproliferative small intestinal disease (Mediterranean lymphoma)	2	0.01	0.00–0.05	0.01	0.00–0.05	2	0.01	0.00–0.02	0.01	0.00–0.02	4	0.01	0.00–0.02	0.01	0.00–0.02
Mature B-cell leukemia			507	0.21	0.19–0.23	0.26	0.24–.29	244	0.10	0.09–0.12	0.11	0.09–0.12	751	0.16	0.15–0.17	0.19	0.17–0.20
	9,833	Prolymphocytic leukemia, B-cell type	42	0.02	0.02–0.03	0.03	0.02–0.04	33	0.02	0.02–0.03	0.03	0.02–0.04	75	0.02	0.02–0.03	0.03	0.02–0.04
	9,940	Hairy cell leukemia	465	0.23	0.21–0.25	0.27	0.25–0.30	211	0.10	0.09–0.12	0.11	0.09–0.12	676	0.16	0.15–0.18	0.19	0.18–0.21
Plasma cell neoplasms			9,075	3.94	3.86–4.02	5.89	5.76–6.02	10,289	3.64	3.57–3.71	4.15	4.07–4.23	19,364	3.78	3.72–3.83	4.97	4.89–5.04
	9,731	Plasmacytoma, NOS	3	0.01	0.00–0.02	0.01	0.00–0.02	1	0.01	0.00–0.03	0.01	0.00–0.03	4	0.01	0.00–0.01	0.01	0.00–0.01
	9,732	Multiple myeloma	7,398	3.21	3.14–3.28	4.84	4.72–4.96	8,508	3.43	3.35–3.50	3.89	3.81–3.97	15,906	3.32	3.27–3.37	4.36	4.29–4.44
	9,733	Plasma cell leukemia	91	0.07	0.06–0.09	0.09	0.07–0.12	112	0.06	0.05–0.07	0.07	0.05–0.08	203	0.06	0.06–0.07	0.08	0.07–0.09
	9,734	Plasmacytoma, extramedullary	1583	0.69	0.65–0.72	0.99	0.94–1.04	1668	0.69	0.65–0.72	0.76	0.72–0.80	3,251	0.69	0.66–0.71	0.88	0.84–0.91
ALL Mature B-cell NHLs			32,408	11.67	11.55–11.80	17.17	16.97–17.37	30,282	10.22	10.10–10.33	11.50	11.37–11.63	62,690	10.92	10.84–11.01	14.33	14.22–14.45

Rates are reported per 100,000 individuals.

*NHL* non-Hodgkin lymphoma, *ICD-O-3* international classification of diseases-oncology-3, *CR* crude rate, *CI* confidence interval, *SR* standardized rate, *CLL/SLL* chronic lymphocytic lymphoma/small lymphocytic leukemia, *NOS* not otherwise specified.

**Figure 2:**
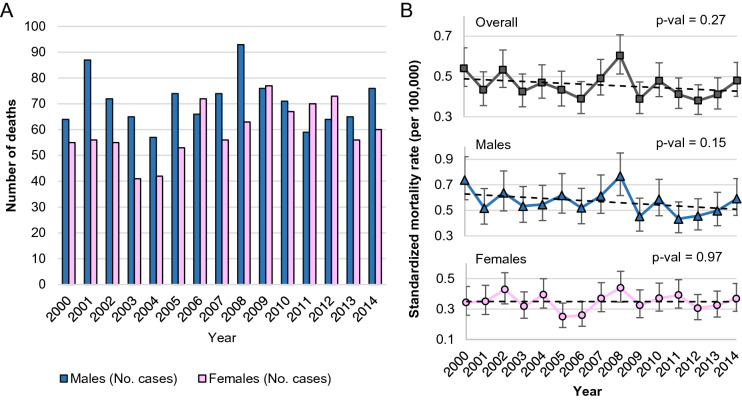
Follicular lymphoma number of deaths and standardized mortality rate, Poland 2000–2014.

In 2014, the standardized death rate in Poland for patients with FL was 0.59/10^5^ for men and 0.37/10^5^ for women. The higher mortality rate for men was observed in the whole follow-up period; in 2000 and 2014, differences in mortality rates between males and females were over 55% and 46%, respectively. The mortality rates for men and women showed no significant trends, and the values of the standardized mortality rate were 0.43–0.76/10^5^ for men and 0.25–0.44/10^5^ for women (see Fig. 2).

Distribution of morphological FL subtypes indicated a high proportion of not otherwise specified (NOS) cases concerning the histological grade; FL NOS accounted for 75% in the male and 70% in the female population. Among the data reported to the NCR, low-grade FL was the most frequently indicated (Grade 1 and Grade 2), and represented 48% and 18% of cases for men and women, respectively. More than one third were FLs with a higher grade of histological malignancy, referred to as Grade 3 (see Supplementary Fig. S2 online).

**Figure 3:**
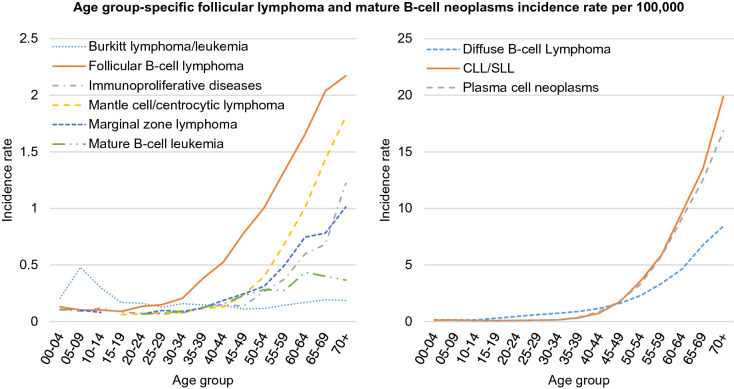
Follicular lymphoma and mature B-cell neoplasms incidence according to age group-specific. *CLL/SLL* chronic lymphocytic leukemia/small lymphocytic lymphoma.

FL was primarily diagnosed in adults, with a median age of 61 years (60 years for men and 61 years for women). According to the NCR data, only about 10% of cases are observed before the age of 40. The highest number of cases in men occurs between 75 and 99 years of age, while in women, between 65 and 74 years of age. The incidence rates for FL are similar in the population of women and men in almost all age groups except in the 65–74 and 75–99 age ranges where the incidence rate is higher in the male population than in the female one by 22% and 41%, respectively. Age group-specific incidence of FL shows almost similar distribution as plasma cell neoplasms; the diffuse B-cell lymphoma, the most frequent mature B-cell NHLs, is observed in patients in more advanced age (> 75 years old) (see Fig. 3).

## Discussion

Global patterns and trends in the FL incidence remain poorly understood since FL cases are registered as part of a wide range of NHLs. According to recent population-based studies, i.e. the GLOBOCAN database, the identification of FL among NHLs is not possible^10^. However, our study, based on the comprehensive NCR data, allowed us to calculate the standardized incidence rate for FL in Poland reliably. We found that within the period 2000–2014, FL was ranked fourth among all diagnosed mature B-cell NHLs and had an incidence rate of 0.87/10^5^. Our analysis is in agreement with the results from the Polish histopathological registry of lymphomas—a non-population wide histopathological data collection managed by the Polish Lymphoma Research Group—where FL comprised only 4.89% of NHLs^3^.

It has been previously argued that the low incidence rates for both lymphoid and myeloid malignancies in Eastern Europe compared to Western Europe could be a result of underreporting^1,2^. In our study, however, the population coverage is 100%, and histopathologically confirmed cases together with the completeness of the registration are over 90%.

In HAEMACARE analysis, the standardized incidence rate for patients up to 54 years from Eastern Europe was found similar to other parts of Europe, whereas lower incidence was observed in the 75- to 99 age group^1^. Our data does not support that observation as the highest number of cases in men occurs between 75 and 99 years of age, while in women, between 65 and 74 years of age. We cannot conclude that the FL incidence is lower due to lower life expectancy in Poland or less frequent diagnostic investigation of the elderly patients.

In addition, high pathological and clinical FL heterogeneity may introduce some case registration errors. There is a possibility that the newly diagnosed patients with high-grade FL “skip” the FL registration because they are usually qualified for the DLBCL, NOS treatment regimen. On the other hand, the low-grade FL cases frequently require only the “watch and wait” strategy, and physicians might neglect to report such cases^11,12^. Moreover, due to indolent behavior and long follow up periods, there can be multiple registrations of the same patient in various local registries. To prevent the latter, registrations are always identified and double-checked at the level of the NCR.

FL incidence differences between populations of Western and Eastern Europe may be associated with variable exposition to known risk factors. The FL prevalence, in contrast to NHLs etiologically associated with EBV, HIV, or HTLV-1 infections, does not reflect any infectious origin^13^. Pesticide exposure measured as lifetime-days of exposure and adjusted risks for NHLs subtypes and FL indicates a significant statistical correlation to lindane—isomer gamma 1,2,3,4,5,6-hexachlorocyclohexane (HCH), a chlorinated hydrocarbon insecticide, which was banned worldwide in 2009—and diazinon—an organophosphate insecticide^14^. After the Second World War, lindan was widely sprayed in Europe and US, and the unwanted by-products (hazardous HCH waste) were discharged at many sites. The release of toxic water-soluble HCH contaminated soil and bioaccumulates via the food chain^15^. According to the latest environmental report, Poland is a country struggling with persistent organic pollutants. However, the scale of contamination was much lower than in other European countries, i.e., Germany, the Netherlands, Spain, France, or Czech Republic^16^.

The large-scale epidemiological analyses showed complex and multifactorial etiology of FL, with a history of cigarette smoking and alcohol consumption being one of the most powerful factors. Cigarette smoking is associated with an increased risk of FL^17,18^. Smoking habits in Eastern European countries are changing dynamically. Currently, Poland is ranked in the top 10 countries around the world with the steepest annual decline in smoking prevalence in both sexes; however, over 8 million Poles are still everyday smokers^19,20^. In contrast, higher alcohol intake is related to a reduced risk of FL; that correlation was confirmed in several epidemiological studies^21,22^. Poland continuously belongs to one of the world leaders in the consumption of pure alcohol, and the alcohol intake maintains an upward trend^23–26^.

The genetic variations, including the major histocompatibility complex class II, i.e., specific haplotypes of human leukocyte antigen, are thought to be strongly related to FL susceptibility^27–29^. Moreover, particular genes polymorphisms might be associated with the elevated risk of lymphoma-specific death, lymphoma progression, or overall survival^30^. There are no extensive molecular and genetic studies for the Poland population; the results include only single-center FL studies with a limited number of cases that can not provide more in-depth epidemiological insight into FL etiopathogenesis^31^.

Our study was conducted in the rituximab era. In 1997 this monoclonal anti-CD20 antibody gained the Food and Drug Administration approval in FL treatment, and in early 2000 the European Union and Poland have started its implementation^32,33^. The standardized death rate in Poland in 2014 is similar to the SEER data^34^. In our analysis, the inferior mortality among males as compared to females is described – the differences are reaching 50%. This phenomenon might be better understood if we consider the fact that the highest number of cases in men occurs between 75 and 99 years, while in women a decade earlier (between 65 and 74 years of age). More advanced age may limit the treatment options due to overlapping comorbidities. Despite the improvement in FL overall survival rate, the leading cause of death is lymphoma-related, especially transformation to DLBCL, NOS^35^. Diminishing of FL mortality is still of great interest and encourages the investigation for less-toxic therapies.

In summary, our study confirms that Poland belongs to a group of countries with a low incidence rate of FL. Our results based on NCR data from over 15 years of observation provides a reliable source of epidemiological FL characteristics. The uprising FL incidence trend in females is marked, but still lower than in Central and Western Europe. The FL mortality remains stable in Poland; its comparison to the other European countries is difficult due to different ways of presenting data. In the near future, we may expect an improvement in the standardized death rate as a result of better prognostics and new therapeutic options. A variety of etiological risk factors and genetic susceptibility should still be investigated for better understanding of FL etiopathogenesis in the Eastern European region.

## Material and methods

The data source for this study was the Polish National Cancer Registry of Poland (NCR), a nationwide, population-based cancer registry with data collected by registration offices in 16 voivodeships. New cases of lymphoma in Poland are documented by cancer registration forms. A double system of codification was applied according to the 10th Revision of the International Classification of Diseases and Related Health Problems (ICD-10) and the 3rd revision of the Classification of Diseases for Oncology (ICD-O-3). The low-grade FL was reported with the 9,695/3 and 9,691/3 codes which were assigned to grade 1 and grade 2, respectively (ICD-10: C82.0 and C82.1); grade 3 (high grade; ICD-O-3: 9,680/3, ICD-10: C82.2) is further subdivided into A (ICD-10: C82.3) and B (ICD-10: C82.4)^36,37^. The percentage of histopathologically confirmed cases and completeness of the registration exceeded 90%.

The Statistics Poland was the source of cancer mortality data (death certificates) and population size and structure of the Polish population by sex and by 5-years age groups for each observation year.

Number of cases, percentages, crude rates, and age/sex standardized rates were calculated based on the revised European Standard Population from 2014^38^. Confidence intervals for crude and standardized incidence rates were calculated using the method based on the gamma distribution. To analyze the significance of trends, we investigated significance (statistical P-value) of Pearson’s product-moment correlation coefficient between year and rate. Statistical analysis was performed using R version 3.6.3 software (R Foundation for Statistical Computing, Vienna, Austria) and *dsr* package implemented therein.

This study was performed according to the Declaration of Helsinki. The study protocol was approved by the Ethical Committee of Maria Sklodowska-Curie National Research Institute of Oncology (reference number: GW23/2017). The Ethical Committee waived written informed consent due to the retrospective nature of the study and the de-identification of the patient information. All methods were carried out following the WHO, ICD-O-3, and ICD-10 guidelines and recommendations^36,37^.

## Supplementary information

Supplementary file 1.

## References

[1] Sant M (2010). Incidence of hematologic malignancies in Europe by morphologic subtype: results of the HAEMACARE project. Blood.

[2] Miranda-Filho A (2019). Global patterns and trends in the incidence of non-Hodgkin lymphoma. Cancer Causes Control CCC.

[3] Szumera-Ciećkiewicz A (2014). Distribution of lymphomas in Poland according to World Health Organization classification: analysis of 11718 cases from National Histopathological Lymphoma Register project: the Polish Lymphoma Research Group study. Int. J. Clin. Exp. Pathol.

[4] Swerdlow SH (2017). WHO classification of tumours of haematopoietic and lymphoid tissues (revised.

[5] Xerri L (2016). The heterogeneity of follicular lymphomas: from early development to transformation. Virchows Archiv. Int. J. Pathol..

[6] Carbone A (2019). Follicular lymphoma. Nat. Rev. Dis. Primers.

[7] de Jong D, de Boer JP (2009). Predicting transformation in follicular lymphoma. Leuk. Lymph..

[8] Fischer T, Zing NPC, Chiattone CS, Federico M, Luminari S (2018). Transformed follicular lymphoma. Ann. Hematol..

[9] Oishi N, Montes-Moreno S, Feldman AL (2018). In situ neoplasia in lymph node pathology. Semin. Diagn. Pathol..

[10] Bray, F.*, et al.* Global cancer statistics 2018: GLOBOCAN estimates of incidence and mortality worldwide for 36 cancers in 185 countries. *CA Cancer J. Clin.* **68**, 394–424 (2018). 10.3322/caac.21492 30207593

[11] Yuda S (2016). Influence of the watch and wait strategy on clinical outcomes of patients with follicular lymphoma in the rituximab era. Ann. Hematol..

[12] Armitage JO, Longo DL (2016). Is watch and wait still acceptable for patients with low-grade follicular lymphoma?. Blood.

[13] Smedby KE, Ponzoni M (2017). The aetiology of B-cell lymphoid malignancies with a focus on chronic inflammation and infections. J. Intern. Med..

[14] Alavanja MCR (2014). Non-hodgkin lymphoma risk and insecticide, fungicide and fumigant use in the agricultural health study. PLoS ONE.

[15] Vijgen J (2011). Hexachlorocyclohexane (HCH) as new Stockholm Convention POPs: a global perspective on the management of Lindane and its waste isomers. Environ. Sci. Pollut. Res. Int..

[16] Vijgen, J., de Borst, B., Weber, R., Stobiecki, T. & Forter, M. HCH and lindane contaminated sites: European and global need for a permanent solution for a long-time neglected issue. *Environ. Pollut. (Barking, Essex : 1987)* **248**, 696–705 (2019). 10.1016/j.envpol.2019.02.029 30849587

[17] Chihara D (2015). New insights into the epidemiology of non-Hodgkin lymphoma and implications for therapy. Expert Rev. Anticancer Ther.

[18] Kroll ME (2012). Alcohol drinking, tobacco smoking and subtypes of haematological malignancy in the UK Million Women Study. Br. J. Cancer.

[19] Smoking prevalence and attributable disease burden in 195 countries and territories, 1990–2015: a systematic analysis from the Global Burden of Disease Study 2015. *Lancet (London, England)* **389**, 1885–1906 (2017). 10.1016/S0140-6736(17)30819-X PMC5439023 28390697

[20] Ng M (2014). Smoking prevalence and cigarette consumption in 187 countries, 1980–2012. JAMA.

[21] Linet MS (2014). Medical history, lifestyle, family history, and occupational risk factors for follicular lymphoma: the InterLymph Non-Hodgkin Lymphoma Subtypes Project. J. Natl. Cancer Inst. Monogr..

[22] Psaltopoulou T (2018). Alcohol consumption and risk of hematological malignancies: a meta-analysis of prospective studies. Int. J. Cancer.

[23] World Health Organization. www.who.int/substance_abuse/publications/global_alcohol_report/profiles/pol.pdf?ua=1.

[24] Wojtyniak B, Moskalewicz J, Stokwiszewski J, Rabczenko D (2005). Gender-specific mortality associated with alcohol consumption in Poland in transition. Addiction (Abingdon, England).

[25] Yakovlev, E. Alcoholism and mortality in Eastern Europe: https://wol.iza.org/articles/alcoholism-and-mortality-in-eastern-europe/long. in *IZA World of Labor* (2015).

[26] WHO Global Status Report On Alcohol and Health. Geneva: WHO, 2011.

[27] Baecklund F (2017). Possible interaction between cigarette smoking and HLA-DRB1 variation in the risk of follicular lymphoma. Am. J. Epidemiol..

[28] Conde L (2010). Genome-wide association study of follicular lymphoma identifies a risk locus at 6p21.32. Nat. Genet..

[29] Skibola CF (2014). Genome-wide association study identifies five susceptibility loci for follicular lymphoma outside the HLA region. Am. J. Hum. Genet..

[30] Baecklund F (2014). A comprehensive evaluation of the role of genetic variation in follicular lymphoma survival. BMC Med Genet.

[31] Paszkiewicz-Kozik E (2009). Presence of t(14;18) positive cells in blood and bone marrow does not predict outcome in follicular lymphoma. Med. Oncol. (Northwood, London, England).

[32] Walewski J (2020). First-line R-CVP versus R-CHOP induction immunochemotherapy for indolent lymphoma with rituximab maintenance: a multicentre, phase III randomized study by the Polish Lymphoma Research Group PLRG4. Br. J. Haematol..

[33] Walewski J (2001). Rituximab (Mabthera, Rituxan) in patients with recurrent indolent lymphoma: evaluation of safety and efficacy in a multicenter study. Med. Oncol. (Northwood, London, England).

[34] National Cancer Intitute. https://seer.cancer.gov/statfacts/html/follicular.html. in The Surveillance, Epidemiology, and End Results

[35] Sarkozy C (2019). Cause of death in follicular lymphoma in the first decade of the rituximab era: a pooled analysis of French and US cohorts. J. Clin. Oncol..

[36] Fritz A (2013). ICD-O-3 terminology approved for use with cases diagnosed January 1, 2014 and after. J. Regis. Manag..

[37] https://icd.who.int/browse10/2016/en#/C81-C96.

[38] Wojciechowska, U., Olasek, P., Czauderna, K. & Didkowska, J. *Nowotwory złośliwe w Polsce w 2014 roku. Cancer in Poland in 2014.*, (Centrum Onkologii Instytut im. Marii Skłodowskiej-Curie, Warszawa 2016).

