# Supplementary material for: Population-based epidemiological data of follicular lymphoma in Poland: 15 years of observation
Source: Sci Rep. 2020 Sep 3;10:14610. doi: 10.1038/s41598-020-71579-6 (PMC7471935; doi:10.1038/s41598-020-71579-6)

**Supplementary Information**

**Title: Population-based epidemiological data of follicular lymphoma in Poland - 15 years of observation.**

**Authors:** Anna Szumera-Ciećkiewicz^1,2^, Urszula Wojciechowska^3^, Joanna Didkowska^3^, Jan Poleszczuk^4,5^, Grzegorz Rymkiewicz^1^, Ewa Paszkiewicz-Kozik^6^, Kamil Sokół^1,2^, Monika Prochorec-Sobieszek^1,2^, Jan Walewski^6^

Affiliations:

1. Department of Pathology and Laboratory Diagnostics, Maria Sklodowska-Curie **National Research Institute of Oncology**, Warsaw, Poland;
2. Department of Diagnostic Hematology, Institute of Hematology and Transfusion Medicine, Warsaw, Poland;
3. Polish National Cancer Registry, Maria Sklodowska-Curie **National Research Institute of Oncology,** Warsaw, Poland;
4. Nalecz Institute of Biocybernetics and Biomedical Engineering, Polish Academy of Sciences, Warsaw, Poland;
5. Department of Computational Oncology, Maria Sklodowska-Curie **National Research Institute of Oncology,** Warsaw, Poland
6. Department of Lymphoid Malignancies, Maria Sklodowska-Curie **National Research Institute of Oncology,** Warsaw, Poland;

Corresponding author:

Anna Szumera-Ciećkiewicz

Maria Sklodowska-Curie **National Research Institute of Oncology,**

Department of Pathology and Laboratory Diagnostics

5 Roentgen Str. 02-781 Warsaw, Poland

e-mail: [szumann@gmail.com](mailto:szumann@gmail.com)

ORCID: 0000-0001-5028-3422

**Supplementary Figure S1.** Morphological subtypes and distribution of mature B-cell NHLs, Poland 2000-2014. CLL/SLL: chronic lymphocytic leukemia/small lymphocytic lymphoma.


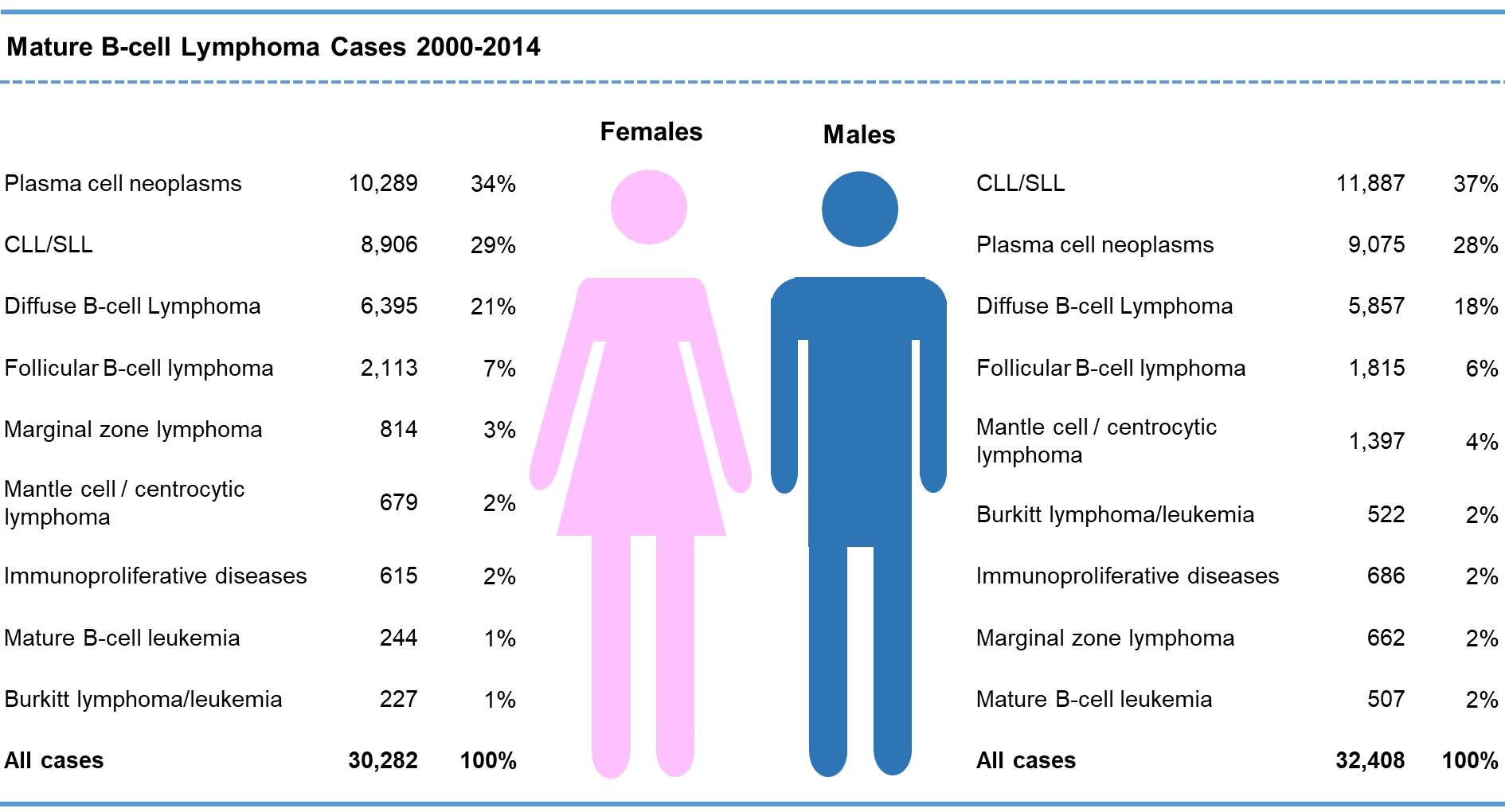


**Supplementary Figure S2.** Follicular lymphoma and histopathological grade distribution, Poland 2000-2014.


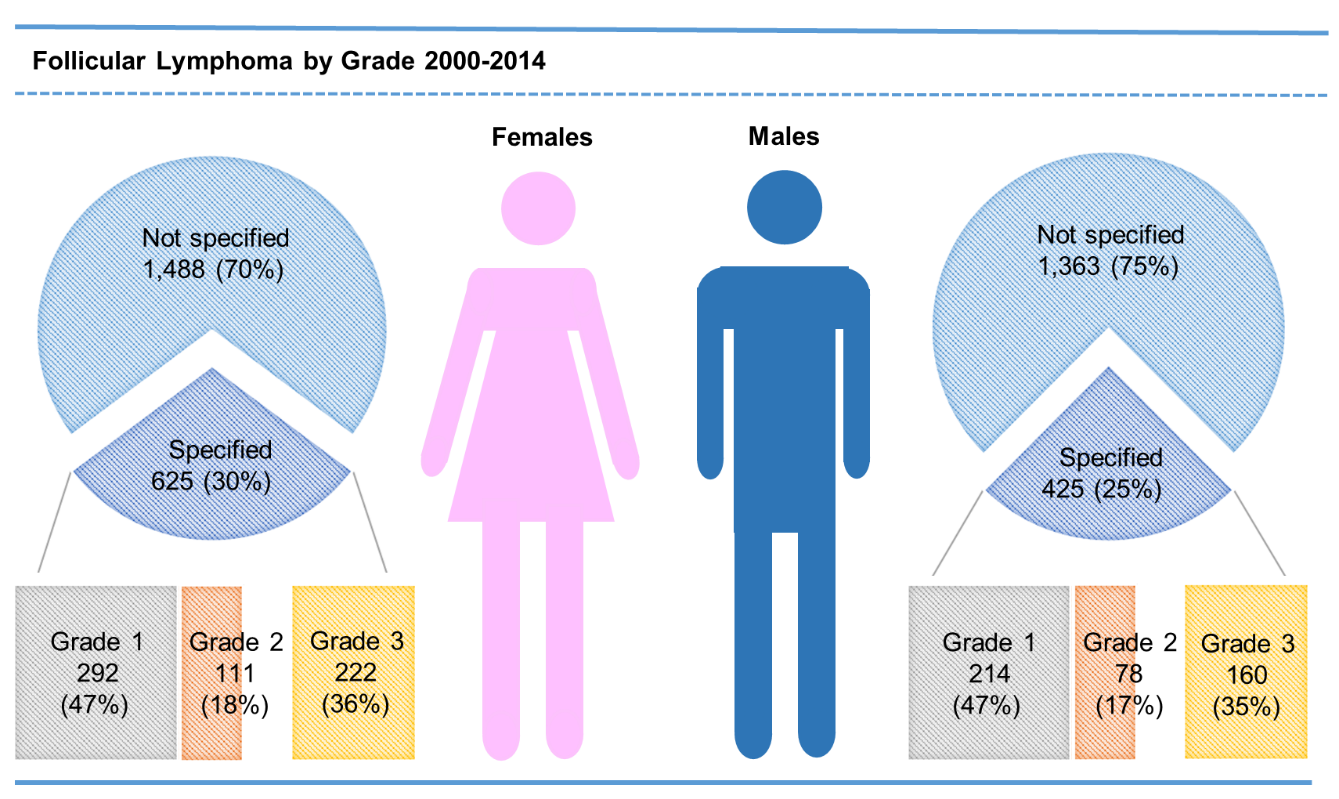

Supplement: Supplementary file 1 [file 41598_2020_71579_MOESM1_ESM.docx]
